# Using serum troponins to screen for cardiac involvement and assess disease activity in the idiopathic inflammatory myopathies

**DOI:** 10.1093/rheumatology/key031

**Published:** 2018-03-12

**Authors:** James B Lilleker, Axel C P Diederichsen, Søren Jacobsen, Mark Guy, Mark E Roberts, Jamie C Sergeant, Robert G Cooper, Louise P Diederichsen, Hector Chinoy

**Affiliations:** 1 Centre for Musculoskeletal Research, School of Biological Sciences, Faculty of Biology Medicine and Health, University of Manchester, Manchester, UK; 2 Greater Manchester Neuroscience Centre, Salford Royal NHS Foundation Trust, Manchester Academic Health Science Centre, Salford, UK; 3 Department of Cardiology, Odense University Hospital, Odense, Denmark; 4 Copenhagen Lupus and Vasculitis Clinic, Center for Rheumatology and Spine Diseases, Rigshospitalet, Copenhagen University Hospital, Copenhagen, Denmark; 5 Department of Biochemistry, Salford Royal NHS Foundation Trust, Manchester Academic Health Science Centre, Salford, UK; 6 Centre for Biostatistics, University of Manchester, Manchester Academic Health Science Centre, Manchester, UK; 7 MRC-ARUK Centre for Integrated Research into Musculoskeletal Ageing, University of Liverpool, Liverpool, UK; 8 Rheumatology Department, Salford Royal NHS Foundation Trust, Manchester Academic Health Science Centre, Salford, UK; 9 Department of Rheumatology, Odense University Hospital, Odense, Denmark; 10 NIHR Manchester Biomedical Research Centre, Manchester University NHS Foundation Trust, Manchester Academic Health Science Centre, Manchester, UK

**Keywords:** autoimmune diseases, cardiovascular disease, dermatomyositis, disease activity, idiopathic inflammatory myopathy, polymyositis

## Abstract

**Objectives:**

Limitations in the methods available for identifying cardiac involvement and accurately quantifying disease activity in the idiopathic inflammatory myopathies (IIMs) may contribute to poor outcomes. We investigated the utility of different serum muscle damage markers [total creatine kinase (CK), cardiac troponin T (cTnT) and cardiac troponin I (cTnI)] to address these issues.

**Methods:**

We assessed disease activity and cardiac involvement using the International Myositis Assessment and Clinical Studies Group core set measures in 123 participants with confirmed adult-onset IIM from the UK and Denmark. Total CK, cTnT and cTnI were measured. Associations were assessed using logistic regression and Spearman’s ranked correlation.

**Results:**

Cardiac involvement (*n* = 18) was associated with higher cTnI levels, independent of overall disease activity [adjusted odds ratio 1.03 (95% CI 1.01, 1.05); *P* = 0.002]. An abnormal cTnI had the highest specificity and positive predictive value for cardiac involvement (95% and 62%, respectively). In those with a normal CK but elevated cTnT or cTnI, an association with increased disease activity scores was observed. Serum cTnT correlated with the physician (ρ = 0.39) and patient-assessed (ρ = 0.28) global visual analogue scales and HAQ (ρ = 0.41) more strongly than CK or cTnI levels. cTnT was the only marker to correlate with manual muscle testing scores (ρ = −0.24).

**Conclusion:**

Serum cTnI testing may have a role in screening for cardiac involvement in IIMs. Abnormal levels of serum cTnT and cTnI are associated with increased disease activity, including in those with a normal CK.

Rheumatology key messages

- In the idiopathic inflammatory myopathies, serum troponin testing may improve disease activity assessments.
- In the idiopathic inflammatory myopathies, abnormal troponin I had the highest specificity for cardiac involvement.
- In the idiopathic inflammatory myopathies, troponin T levels correlated most strongly with disease activity.

## Introduction

The idiopathic inflammatory myopathies (IIMs) are multisystem autoimmune diseases characterized primarily by skeletal muscle inflammation. Limitations in current methods for detecting early signs of organ-specific flares can delay timely commencement or escalation of treatment and may contribute to poor outcomes. Disease activity in IIMs can be quantified using the International Myositis Assessment and Clinical Studies Group (IMACS) core set measures (CSMs). These include the measurement of different serum muscle damage markers (“muscle enzymes”), total creatine kinase (CK), lactate dehydrogenase, aldolase or transaminases [1]. As total CK can be normal in patients with active IIM, the isolated use of this parameter can be misleading, but it remains common practice [2].

Cardiac involvement occurs in up to 75% of patients with IIM, is often subclinical, represents a major cause of morbidity and mortality and is sometimes only identified post-mortem [3]. Several methods are used in clinical practice to screen for cardiac involvement in IIM, including electrocardiography, echocardiography and contrast-enhanced cardiac MRI [4]. So called cardiac-specific serum muscle damage markers [cardiac troponin T (cTnT) and cardiac troponin I (cTnI)] may have a role, but can lack specificity for cardiac disease in IIM, especially the cTnT, which is often elevated in those with skeletal muscle disease without cardiac involvement [5, 6].

In this cross-sectional study, we evaluated the usefulness of serum cTnT and cTnI levels in screening for cardiac involvement and in quantifying disease activity in patients with IIM. Comparison is made with the use of serum total CK and the potential for using cTnT or cTnI as the second serum muscle damage marker in the IMACS disease activity CSMs assessment.

## Methods

### Cases

We performed an analysis of data pooled from two cohorts, one in the UK and one in Denmark (*n* = 123). Data regarding some of the Danish cohort has been previously published as part of separate studies [4, 7]. Details of patient identification procedures at each site are contained in the supplementary data, section ‘Patient identification’, available at *Rheumatology* online.

### Assessment

Each case was assessed using the IMACS disease activity CSMs. This included manual muscle testing-8 (MMT-8), HAQ disability index (HAQ-DI), physician and patient-assessed global disease activity visual analogue scales (VASs) and an extramuscular disease assessment using the Myositis Disease Activity Assessment Tool (MDAAT) [1].

Cases also supplied blood for measurement of serum total CK, cTnT and cTnI (supplementary data, section ‘Laboratory assays’, available at *Rheumatology* online). The results were not available to clinicians at the time of their performing clinical assessments. Normalization of CK and cTnI levels was performed to allow comparison of results from different assays (supplementary data, section ‘Laboratory assays’, available at *Rheumatology* online). A result was deemed abnormal if the value was above the upper limit of the laboratory reference range (for cTnT) or normalized reference range (for CK and cTnI).

### Cardiac involvement

Cardiac involvement was determined as per the cardiac domain of the MDAAT, the agreed upon tool for evaluating the presence and extent of extramuscular involvement in IIM [8]. The MDAAT has been partially validated, performs well in terms of interrater reliability and is used in IIM clinical trials, including the recently published Rituximab in Myositis study [9]. In the current study, the assessment could include consideration of any recently performed cardiac investigations (e.g. electrocardiography or echocardiogram), but additional investigations were not performed. Cardiac involvement was deemed as present when the cardiac VAS score on the MDAAT was >0.

### Statistical analysis

Data processing and analysis was performed using STATA for Windows version 13.0 (StataCorp, College Station, TX, USA). Normally distributed data were summarized by calculation of means and standard deviations (s.d.). Non-normally distributed data were summarized using medians and interquartile ranges (IQRs). Convergent construct validity of the serum muscle damage markers was assessed by Spearman’s ranked correlation with other aspects of disease activity. A correlation coefficient (ρ) >0.70 was considered as representing a strong relationship, 0.25–0.70 a moderate relationship and <0.25 a weak relationship. Logistic regression was used to assess associations with cardiac involvement and abnormal serum muscle damage markers. All models were adjusted for cohort (i.e. UK or Denmark). The physician-completed global disease activity VAS was used to adjust for overall IIM disease activity where specified. *P*-values <0.05 were considered significant.

### Ethics and consent

Full informed consent was obtained from all participating patients. The study was completed in full conformance with the principles of the Declaration of Helsinki. In the UK, ethical approval was obtained from the Health Research Authority (East Midlands – Leicester Central Research Ethics Committee, reference 14/EM/1248). We also conformed to Good Clinical Practice and the laws and regulations of the UK. In Denmark, the study was approved by the local ethics committee (reference S-20100022).

## Results

A total of 123 cases were analysed (Denmark 79, UK 44; supplementary Table S1, available at *Rheumatology* online). Overall, 32% (39/123) of cases had DM, 28% (34/123) had PM, 30% (37/123) had anti-synthetase syndrome, 7% (8/123) had immune-mediated necrotizing myopathy and 4% (5/123) had IIM-CTD overlap disease (supplementary Table S2, available at *Rheumatology* online). The mean age was 58 years (s.d. 14), 66% (81/123) of cases were female and 94% (116/123) were Caucasian.

### Screening for cardiac involvement

Eighteen cases were deemed to have cardiac involvement [15% (18/121); MDAAT not completed in two cases] (Table 1). Higher disease activity levels (physician global disease activity VAS, HAQ and extramuscular global VAS) were associated with cardiac involvement. No difference in diagnostic subtype, serotype, age, disease duration, gender or smoking status between those with or without cardiac involvement was detected.

**Table 1:** Disease activity and serum muscle damage marker levels stratified by the presence of cardiac involvement

	Cardiac involvement (n = 18)^a^	No cardiac involvement (n = 103)	Odds ratio	*P*-value
Diagnostic subcategory, *n* (%)				
DM	5 (28)	34 (33)	0.79 (0.19, 3.20)	0.736
Anti-synthetase syndrome	6 (33)	29 (28)	1.15 (0.31, 4.28)	0.655
PM	5 (28)	29 (28)	Referent	—
IMNM	2 (11)	6 (6)	1.62 (0.21, 12.72)	0.698
Myositis–CTD overlap	0 (0)	5 (5)	1	—
Autoantibody profile, *n* (%)				
Myositis-specific antibody	11 (61)	49 (48)	2.25 (0.57, 8.92)	0.249
Myositis-associated antibody	4 (22)	24 (23)	1.67 (0.34, 8.24)	0.530
Seronegative	3 (17)	30 (29)	Referent	—
Disease duration, median (IQR), years	2.8 (0.4–12)	5.2 (1.3–10)	0.96 (0.87, 1.06)	0.412
Age, mean (s.d.), years	58 (18)	58 (13)	1.00 (0.97, 1.04)	0.879
Female gender, *n* (%)	12 (67)	67 (65)	1.08 (0.37, 3.08)	0.909
Ever smoked, *n* (%)	9 (50)	44 (43)	1.40 (0.50, 3.93)	0.521
Disease activity measures, median (IQR)				
Physician global disease activity VAS	3.2 (1.0–6.5)	1.4 (0.8–3.3)	1.24 (1.03, 1.49)	**0.021**
Patient global disease activity VAS	5.2 (1.7–7.9)	4.3 (2.2–6.1)	1.12 (0.91, 1.38)	0.291
HAQ-DI	1.6 (0.4–2.4)	0.5 (0–1.3)	2.69 (1.42, 5.07)	**0.002**
Extramuscular global VAS^c^	2.2 (0.6–4.0)	0.9 (0.4–1.9)	1.30 (1.05, 1.61)	**0.017**
Manual muscle testing	76 (68–79)	77 (73–79)	0.93 (0.85, 1.01)	0.074
Serum muscle damage markers, median (IQR)				
Total CK, IU/L^d^	497 (95–771)	134 (81–331)	1.00 (0.99, 1.00)	0.465
Abnormal total CK, *n* (%)	11 (61)	37 (36)	Sensitivity 61% Specificity 64% PPV 23%	
cTnT, ng/l	91 (29–156)	17 (8–48)	1.00 (1.00, 1.01)	**0.013**
Abnormal cTnT, *n* (%)	15 (83)	56 (54)	Sensitivity 83% Specificity 46% PPV 21%	
cTnI, ng/l^d^	25 (5–128)	5 (0–16)	1.03 (1.01, 1.05)	**0.001**
Abnormal cTnI, *n* (%)	8 (44)	5 (5)	Sensitivity 44% Specificity 95% PPV 62%	

Cardiac involvement was defined as a score >0 on the cardiac VAS component of the MDAAT. Abnormal levels of serum muscle damage markers are defined as a value greater than the upper limit of the laboratory reference range. Two cases are omitted from this table because the cardiac domain of the MDAAT was not completed. ORs are adjusted for country of origin of the participants and are derived from logistic regression. Bold values indicate P < 0.05.

a Eighteen patients had cardiac involvement. Using categories defined in the MDAAT with the following frequencies: myocarditis, 12; sinus tachycardia, 6; arrhythmia, 4; pericarditis, 1 (some patients had multiple types).

b Adjusted for cohort (i.e. UK or Denmark).

c A component of the MDAAT.

d Normalized values shown.

IMNM: immune-mediated necrotizing myopathy.

The cTnI level correlated most strongly with the cardiac disease activity VAS (Table 2). An increased risk of cardiac involvement was associated with increasing serum levels of cTnT and cTnI, although this was independent of overall disease activity only for cTnI [adjusted odds ratio (OR) 1.03 (95% CI 1.01, 1.05), *P* = 0.002].

**Table 2:** Correlation (ρ) of serum muscle damage marker levels with each other and disease activity

	Total CK^a^	cTnT	cTnI^a^
cTnT	0.61**	—	—
cTnI	0.20*	0.44**	—
Physician global disease activity VAS	0.21*	0.39**	0.22*
Patient global disease activity VAS	0.21*	0.28*	0.22*
HAQ-DI	0.28*	0.41**	0.29*
Extramuscular global VAS^b^ (*n* = 121)	−0.03	0.16	0.24*
Cardiac activity VAS^b^ (*n* =121)	0.15	0.31**	0.35**
Manual muscle testing	0.14	−0.24*	−0.12

a Normalized values used.

b Components of the MDAAT.

* *P* < 0.05;

** *P* < 0.001.

ρ: Spearman’s rank correlation coefficient.

An abnormal cTnI had the highest specificity and positive predictive value (PPV) for cardiac involvement but the lowest sensitivity (Table 1). Cardiac involvement was only present in 23% (11/48) of those with an abnormal total CK and 21% (15/71) of those with an abnormal cTnT, compared with 62% (8/13) of those with an abnormal cTnI. When stratifying serum muscle damage marker levels according to the type of cardiac involvement identified (as per the MDAAT: myocardial, pericardial, arrhythmia or sinus tachycardia), only myocardial disease was associated with increasing levels of cTnT and cTnI [for cTnT: adjusted OR 1.00 (95% CI 1.00, 1.01), *P* = 0.006; for cTnI: adjusted OR 1.01 (95% CI 01.00, 1.02), *P* = 0.015] (supplementary Table S3, available at *Rheumatology* online).

### Correlation between serum muscle damage marker levels and disease activity

The cTnT levels correlated most strongly with the physician global VAS, the patient global VAS, the HAQ disability index (HAQ-DI) and the manual muscle testing score, outperforming CK and cTnI in all cases (Table 2). cTnT was the only serum marker to correlate with the MMT-8 score and cTnI was the only marker to correlate with the extramuscular global VAS. The total CK level had the weakest correlations with the physician global VAS, the patient global VAS and the HAQ-DI and showed no significant correlation with the extramuscular global VAS, the cardiac disease VAS or the MMT-8.

### The added value of troponin testing

In 40% (29/73) of cases that had a normal total CK, the cTnT was elevated. In these cases, the abnormal cTnT was associated with lower MMT-8 scores [median 74 (IQR 72–78) *vs* 78 (75–79); adjusted OR 0.83 (95% CI 0.71, 0.96); *P* = 0.011] and higher patient-assessed global disease activity VAS [4.8 (IQR 2.7–7.4) *vs* 2.6 (1.8–5.4); adjusted OR 1.30 (95% CI 1.04, 1.62); *P* = 0.019]. Despite the elevated cTnT, an increased risk of cardiac involvement was not seen when compared with those with normal cTnT [14% (4/29) *vs* 7% (3/44); adjusted OR 2.20 (95% CI 0.45, 10.78); *P* = 0.332].

The cTnI was elevated in 7% (5/73) of those with a normal total CK. In such cases, an abnormal cTnI was associated with increasing patient-assessed global disease activity VAS [median 7.8 (IQR 7.3–8.1) *vs* 3.3 (1.9–5.5); adjusted OR 2.54 (95% CI 1.25, 5.18); *P* = 0.010], and with cardiac involvement [29% (2/7) *vs* 5% (3/66); adjusted OR 10.36 (95% CI 1.22, 87.72); *P* = 0.032].

## Discussion

### Interpretation and implications

The cTnI levels were significantly increased in those with cardiac involvement and this was independent of overall disease activity. An abnormal cTnI had the highest PPV for cardiac involvement and was the only damage marker to correlate significantly with the extramuscular global VAS. This suggests that the cTnI level provides information about a different facet of IIM disease activity compared with CK and cTnT and is perhaps most useful as a screening tool for cardiac involvement. However, the low sensitivity for cardiac involvement implies that a normal cTnI level does not exclude it. In contrast, the low specificity of cTnT and CK for cardiac involvement may generate unnecessary concern about such involvement. Those with cardiac involvement had significantly worse overall disease activity, suggesting that clinicians should have a low threshold for considering the potential for cardiac involvement in patients with highly active IIM.

We have demonstrated that, of the muscle damage markers tested, cTnT levels correlated most strongly with IIM disease activity. We also identified significantly worse aspects of disease activity in those with normal CK but abnormal cTnT or cTnI, confirming the notion that the use of CK in isolation to assess IIM disease activity can be misleading.

Elevations of cTnT and the CK-MB fraction (both conventionally used as cardiac damage markers) are observed frequently in those with IIM in the absence of cardiac involvement [10–12]. Potential explanations for this include the existence of subtle subclinical cardiac involvement, as supported by time–kinetic analysis, which suggests that elevated cTnT in IIM is of cardiac origin in at least some cases [13].

Cross-reactivity of the cTnT assay with skeletal muscle troponin T (sTnT) is another possible explanation for elevations seen in IIM. However, very low levels of cross-reactivity are reported for the current generation of assays [12, 14]. Finally, it has been shown that regenerating skeletal muscle fibres can express cTnT and CK-MB, thus elevations in IIM may actually originate from skeletal muscle undergoing repair rather than from myocardium [5, 14, 15]. Importantly, as cTnI does not share this characteristic, elevated levels should be assumed to have arisen from the myocardium [16, 17].

### Strengths and limitations

This study included thorough assessment of cases using the IMACS disease activity CSMs toolkit and is the largest study to use this panel of serum muscle damage markers. Researchers were blinded to serum muscle damage marker levels during patient clinical assessments, thus minimizing the potential for bias. However, there are several limitations. The study lacked a gold standard to define cardiac involvement, relying on assessment using the partially validated MDAAT, without additional investigations being performed. However, during phase two of the MDAAT validation study, the sensitivity, specificity and PPV for active cardiac disease was 100% [8]. Despite this, our study may underestimate the presence of cardiac involvement, particularly subtle subclinical disease. It is also possible that subtle abnormalities on any investigations performed may not have been identified. Future studies may be improved by the use of skeletal muscle and cardiac MRI to understand the relative contributions to serum muscle damage marker elevations and the relationship between subclinical (e.g. purely radiologically evident) cardiac involvement and serum muscle damage marker elevations in IIM. While we did not include analysis of the intent-to-treat aspect of the MDAAT cardiac assessment, high correlation has been demonstrated between this and the severity assessment (*r* = 0.94) [8].

As data were pooled from separate studies, this creates the possibility of systematic bias influencing the results due to differing practices in each centre. However, similar proportions of cardiac involvement were identified in both cohorts [16% (7/44) in the UK and 14% (11/77) in Denmark]. We reported the PPV for cardiac involvement (i.e. the probability that subjects with an abnormal serum muscle damage marker truly have the cardiac involvement). Therefore, in settings where the prevalence of cardiac involvement and may differ, the PPV we defined may be less applicable.

### Conclusion

We have demonstrated the potential benefits of measuring serum cTnI and cTnT levels in patients with IIM, enhancing the disease activity assessment and assisting with the identification of cardiac involvement.

Where an abnormal cTnI is encountered in IIM, this should alert one to the possibility of cardiac involvement. In such cases, and in patients with highly active IIM, clinicians should have a low threshold for arranging further investigations to evaluate this possibility. In cases with a normal CK but abnormal cTnT levels, significantly increased levels of disease activity were identified without an increased frequency of cardiac involvement.

## Supplementary Material

Supplementary Data

## References

[1] Rider LG, Werth VP, Huber AM Measures of adult and juvenile dermatomyositis, polymyositis, and inclusion body myositis: Physician and Patient/Parent Global Activity, Manual Muscle Testing (MMT), Health Assessment Questionnaire (HAQ)/Childhood Health Assessment Questionnaire (C-HAQ). Arthritis Care Res 2011;63(Suppl 1):S118–57. 10.1002/acr.20532 PMC3748930 22588740

[2] Miller FW. New approaches to the assessment and treatment of the idiopathic inflammatory myopathies. Ann Rheum Dis 2012;71(Suppl 2):i82–5. 22460145 10.1136/annrheumdis-2011-200587

[3] DeVere R, Bradley WG. Polymyositis: its presentation, morbidity and mortality. Brain 1975;98:637–66. 1218371 10.1093/brain/98.4.637

[4] Diederichsen LP, Simonsen JA, Diederichsen AC Cardiac abnormalities in adult patients with polymyositis or dermatomyositis as assessed by noninvasive modalities. Arthritis Care Res 2016;68:1012–20. 10.1002/acr.22772 26502301

[5] Kiely PD, Bruckner FE, Nisbet JA, Daghir A. Serum skeletal troponin I in inflammatory muscle disease: relation to creatine kinase, CKMB and cardiac troponin I. Ann Rheum Dis 2000;59:750–1. 11023449 10.1136/ard.59.9.750 PMC1753274

[6] Aggarwal R, Lebiedz-Odrobina D, Sinha A, Manadan A, Case JP. Serum cardiac troponin T, but not troponin I, is elevated in idiopathic inflammatory myopathies. J Rheumatol 2009;36:2711–4. 19833747 10.3899/jrheum.090562

[7] Lilleker JB, Guy M, Roberts ME Serum muscle damage markers in the idiopathic inflammatory myopathies: quantifying disease activity and identifying cardiac involvement. Neuromuscul Disord 2017;27:S39–40.

[8] Sultan SM, Allen E, Oddis CV Reliability and validity of the myositis disease activity assessment tool. Arthritis Rheum 2008;58:3593–9. 18975333 10.1002/art.23963

[9] Oddis CV, Reed AM, Aggarwal R Rituximab in the treatment of refractory adult and juvenile dermatomyositis and adult polymyositis: a randomized, placebo-phase trial. Arthritis Rheum 2013;65:314–24. 23124935 10.1002/art.37754 PMC3558563

[10] Rider LG, Miller FW. Laboratory evaluation of the inflammatory myopathies. Clin Diagn Lab Immunol 1995;2:1–9. 7719899 10.1128/cdli.2.1.1-9.1995 PMC170091

[11] Schwarzmeier JDJ, Hamwi A, Preisel M Positive troponin T without cardiac involvement in inclusion body myositis. Hum Pathol 2005;36:917–21. 16112010 10.1016/j.humpath.2005.06.009

[12] Erlacher P, Lercher A, Falkensammer J, Nassonov E. Cardiac troponin and β-type myosin heavy chain concentrations in patients with polymyositis or dermatomyositis. Clin Chim Acta 2001;306:27–33. 11282091 10.1016/s0009-8981(01)00392-8

[13] Fisher C, Agrawal S, Wong WM, Fahie-Wilson M, Dasgupta B. Clinical observations on the significance of raised cardiac troponin-T in patients with myositis of varying etiologies seen in rheumatology practice. Clin Rheumatol 2010;29:1107–11. 20556454 10.1007/s10067-010-1511-6

[14] Bodor GS, Survant L, Voss EM Cardiac troponin T composition in normal and regenerating human skeletal muscle. Clin Chem 1997;43:476–84. 9068591

[15] Lott JA, Abbott LB. Creatine kinase isoenzymes. Clin Lab Med 1986;6:547–76. 3527543

[16] Hughes M, Lilleker JB, Herrick AL, Chinoy H. Cardiac troponin testing in idiopathic inflammatory myopathies and systemic sclerosis-spectrum disorders: biomarkers to distinguish between primary cardiac involvement and low-grade skeletal muscle disease activity. Ann Rheum Dis 2015;74:795–8. 25732174 10.1136/annrheumdis-2014-206812 PMC4589894

[17] Bodor GS, Porterfield D, Voss EM, Smith S, Apple FS. Cardiac troponin-I is not expressed in fetal and healthy or diseased adult human skeletal muscle tissue. Clin Chem 1995;41:1710–5. 7497610

