# Supplementary material for: Using serum troponins to screen for cardiac involvement and assess disease activity in the idiopathic inflammatory myopathies
Source: Rheumatology (Oxford). 2018 Mar 12;57(6):1041–6. doi: 10.1093/rheumatology/key031 (PMC5965080; doi:10.1093/rheumatology/key031)
Supplement: Supplementary Data [file key031_rhe-17-1391-file002.docx]

## Supplementary data

### Patient Identification

#### United Kingdom

Cases were identified from tertiary adult IIM clinics at Salford Royal NHS Foundation Trust (UK) between February 2015 and October 2016. Patients with dermatomyositis (DM) and polymyositis (PM) met criteria for definite or probable idiopathic inflammatory myopathy (IIM) using the Bohan and Peter (B&P) diagnostic criteria, and had a minimum diagnostic probability cut-off ≥50% using the International Myositis Classification Criteria Project (IMCCP) draft diagnostic criteria.^1–3^ We used the term myositis-connective tissue disesae (CTD) overlap for patients that these criteria, that had a co-exisiting CTD. Those with anti-synthetase syndrome had myositis meeting the B&P or IMCCP criteria and the presence of a serum anti-synthetase autoantibody.^4^ Patients with immune-mediated necrotising myopathy met ENMC criteria.^5^ Patients were excluded if an alternative explanation for elevations in serum muscle damage markers was known prior to assessment (e.g. renal impairment, unstable ischaemic heart disease etc.).

#### Denmark

For this study we used data from a recently project undertaken in Denmark.^6^ In summary, patients diagnosed with PM or DM between 2000 and 2010 were identified from the Danish National Patient Register (International Classification of Diseases, Tenth Revision, codes M33.1, M33.2, and M33.9). All Danish patients are recorded within this nationwide register that contains various health-related outcomes. Medical records of the identified patients from 3 regions (populated by approximately 2.1 million inhabitants) out of 5 regions in Denmark were reviewed and patients age ≥18 years fulfilling the Bohan and Peter criteria definite or probable were invited to participate in the study.^2,3^ In this cohort, patients with a co-existing CTD were excluded. Upon review, one patient was also found to meet criteria for immune mediated necrotising myopathy and was reclassified as such.^5^ In accordance with criteria suggested by Connors *et al* we reclassified patients as anti-synthetase syndrome where the presence of an anti-synthetase autoantibody was detected.^4^

### Laboratory assays

#### Serum total creatine kinase (CK)

In the UK, serum total CK was measured using the N-Acetyl Cysteine (NAC) activated International Federation of Clinical Chemistry (IFCC) method on Siemens Advia analysers (reference range 40-320 U/L for males, 25-200 U/L for females). For the Danish cases, serum total CK was measured at different hospitals throughout the country with different assays. To allow comparison all values were normalised (see below).

#### Serum cardia troponin T (cTnT)

For both countries, serum cTnT was measured using the Elecsys hs-TnT assay (chemiluminescent immunoassay) on a Cobas Roche 8000 analyser (reference range ≤14 ng/L).

#### Serum cardia troponin I (cTnI)

In the UK, serum cTnI was measured using the TnI-Ultra assay (chemiluminescent immunoassay) on Siemens Advia Centaur analyser (reference range ≤40 ng/L). In Denmark, serum cTnI was measured using the ARCHITECT STAT Troponin-I assay from Abbott (reference range ≤25 ng/L). To allow comparison all values were normalised (see below).

#### Abnormal levels

Where a measurement result was outside the detectable range of the assay, a value of one point above or below the maximum or minimum detectable value was assigned respectively. The result of a serum muscle damage marker was deemed abnormal if the value was above the upper limit of the reference range.

#### Normalisation procedure

The CK and cTnI levels were normalised prior to analysis. First an arbitrary upper limit value (210 IU/L for CK and 40 ng/l for cTnI) was divided by the upper limit of the laboratory reference range for the individual assay used. This result was then multiplied by the original laboratory result to give the normalised value.

**Supplementary Table S1: Comparison of case characteristics by country**

|  | Denmark cohort (n=79) | UK cohort (n=44) |
| --- | --- | --- |
| **Diagnostic subcategory**  *Dermatomyositis*  *Anti-synthetase syndrome*  *Polymyositis*  *Immune-mediated necrotising myopathy*  *Myositis-CTD overlap* | 27% (21/79)  34% (27/79)  38% (30/79)  1% (1/79)  0 | 41% (18/44)  23% (10/44)  9% (4/44)  16% (7/44)  11%(5/44) |
| Age in years at assessment, mean (SD) | 61 (12) | 52 (16) |
| Disease duration in years at assessment, median (IQR) | 6 (3.9-12.7) | 2 (0.4-6.1) |
| **Gender, %**  male  female | 3 (29/79)  63 (50/79) | 30% (13/44)  71 (31/44) |
| **Ethnicity, n (%)**  White Caucasian Asian  Black | 98 (77/79)  0  3 (2/79) | 89 (39/44)  5 (2/44)  7 (3/44) |
| Ever smoked, n (%) | 52% (41/79) | 30% (13/44) |
| **Disease activity, median (IQR)**  Physician global disease activity VAS, 0-10  Patient global disease activity VAS, 0-10  HAQ disability index, 0-3  Manual muscle testing, 0-80  Extramuscular global VAS, 0-10 | 1.44 (0.89-3.44)  5.05 (2.4-8.1)  0.38 (0-1.19)  75 (72-78)  1.0 (0.60-2.60) | 1.97 (0.64-3.88)  3.35 (1.70-5.48)  1.13 (0.25-2.13)  79 (77-80)  0.75 (0.32-2.34) |
| **Serum muscle damage markers, median (IQR)**  Total creatine kinase^a^  Cardiac troponin T  Cardiac troponin I^a^ | 134 (93-322)  17 (8-48)  11 (0-19) | 183 (65-692)  26 (10-91)  5 (5-10) |
| Cardiac involvement (n=121) | 14% (11/77) | 16% (7/44) |

^a^normalised values shown

**Table s2: Baseline characteristics** **of study participants**

| **Case characteristics (n=123)** | | |
| --- | --- | --- |
| **Diagnostic subcategory, n (%)** |  |  |
| *Dermatomyositis*  *Anti-synthetase syndrome*  *Polymyositis*  *Immune-mediated necrotising myopathy*  *Myositis-CTD overlap* | 39 (32)  37 (30)  34 (28)  8 (7)  5 (4) |  |
| Myositis specific antibody present, n (%)^a^ | 66 (54) |  |
| Age in years at assessment, mean (SD) | 58 (14) |  |
| Disease duration in years at assessment, median (IQR) | 4.3 (1.1-10.2) |  |
| Gender, n (%)  male  female | 42 (34)  81 (66) |  |
| **Ethnicity, n (%)**  White Caucasian Asian  Black | 116 (94)  2 (2)  5 (4) |  |
| Ever smoked, n (%) | 54 (44) |  |
| **Disease activity, median (IQR)**  Physician global disease activity VAS, 0-10  Patient global disease activity VAS, 0-10 (n=118)  HAQ disability index, 0-3 (n=120)  Manual muscle testing, 0-80  Extramuscular global VAS^b^, 0-10 (n=122) | 1.6 (0.8-3.7)  4.3 (2.2-6.4)  0.6 (0.1-1.5)  77 (73-79)  1.0 (0.5-2.6) |  |
| **Serum muscle damage markers, median (IQR)**  Total creatine kinase (ULN=210 IU/L)^c^  Cardiac troponin T (ULN=14 ng/L)  Cardiac troponin I (ULN=40 ng/L)^c^ | 146 (89-475)  20 (9-64)  5 (2-16) |  |
| Cardiac involvement, n (%) | 18 (15) |  |

Cardiac involvement was defined as a cardiac VAS score on the MDAAT >0. ^a^anti-Jo-1=30, anti-TIF1-gamma=6, anti-SRP=10, anti-NXP2=5, anti-SAE=2, anti-HMGCR=2, anti-Mi2=4, anti-MDA5=1, anti-PL-7=5, anti-PL-12=3, anti-EJ=3 (5 patients had dual positivity). ^b^from the Myositis Disease Activity Assessment Tool (MDAAT). ^c^normalised values. IQR: interquartile range; VAS: visual analogue scale.

**Supplementary Table S3: Types of cardiac involvement observed in 18 patients and associated serum muscle damage marker abnormalities**

| Type of cardiac involvement^a^ | Frequency | Abnormal CK (%) | Abnormal cTnT (%) | Abnormal cTnI (%) |
| --- | --- | --- | --- | --- |
| Myocarditis | 12 | 8 (67) | 10 (83) | 8 (67) |
| Pericarditis | 1 | 1 (100) | 1 (100) | 1 (100) |
| Arrhythmia | 4 | 1 (25) | 3 (75) | 2 (50) |
| Sinus tachycardia | 6 | 3 (50) | 5 (83) | 1 (17) |

Five patients had two types of cardiac involvement identified. ^a^As defined using the Myositis Disease Activity Assessment Tool (MDAAT) and associated glossary.

## References

1 Lundberg IE, Miller FW, Tjärnlund A, Bottai M. Diagnosis and classification of idiopathic inflammatory myopathies. *J Intern Med* 2016;**280**:39–51.

2 Bohan A, Peter JB. Polymyositis and Dermatomyositis. *N Engl J Med* 1975;**292**:344–7.

3 Bohan A, Peter JB. Polymyositis and dermatomyositis (second of two parts). *N Engl J Med* 1975;**292**:403–7.

4 Connors GR, Christopher-Stine L, Oddis C V., Danoff SK. Interstitial lung disease associated with the idiopathic inflammatory myopathies: what progress has been made in the past 35 years? *Chest* 2010;**138**:1464–74.

5 Hoogendijk JE, Amato AA, Lecky BR, Choy EH, Lundberg IE, Rose MR, *et al.* 119th ENMC international workshop: trial design in adult idiopathic inflammatory myopathies, with the exception of inclusion body myositis, 10-12 October 2003, Naarden, The Netherlands. *Neuromuscul Disord* 2004;**14**:337–45.

6 Diederichsen LP, Simonsen JA, Diederichsen AC, Hvidsten S, Hougaard M, Junker P, *et al.* Cardiac Abnormalities in Adult Patients With Polymyositis or Dermatomyositis as Assessed by Noninvasive Modalities. *Arthritis Care Res (Hoboken)* 2016;**68**:1012–20.
